# A population-based sex-stratified study to understand how health status preceding traumatic brain injury affects direct medical cost

**DOI:** 10.1371/journal.pone.0240208

**Published:** 2020-10-13

**Authors:** Vincy Chan, Mackenzie Hurst, Tierza Petersen, Jingqian Liu, Tatyana Mollayeva, Angela Colantonio, Mitchell Sutton, Michael D. Escobar

**Affiliations:** 1 KITE-Toronto Rehab, University Health Network, Toronto, Ontario, Canada; 2 Dalla Lana School of Public Health, University of Toronto, Toronto, Ontario, Canada; 3 Rehabilitation Sciences Institute, University of Toronto, Toronto, Ontario, Canada; 4 ICES, Toronto, Ontario, Canada; 5 Department of Occupational Science and Occupational Therapy, University of Toronto, Toronto, Ontario, Canada; University Hospital Zurich, SWITZERLAND

## Abstract

**Objective:**

To understand how pre-injury health status present five-years preceding traumatic brain injury (TBI) affects direct medical cost two years post-injury.

**Methods:**

Patients age ≥19 years in the emergency department (ED) or acute care for a TBI between April 1, 2007 and March 31, 2014 in Ontario, Canada (N = 55,669) were identified from population-based health administrative data. Forty-three factors of pre-injury health status (i.e., comorbidities and personal, social, and environmental factors) that were internally validated for the TBI population were assessed in this study. The outcome of interest was direct medical cost within two years of discharge. Sex-specific multivariable linear regressions were conducted to understand the associations between direct medical cost within two years of discharge and pre-injury health status.

**Results:**

Patients who received care in the ED (81.9% of total sample) incurred a median cost of $2,492/male patient (average $12,342/patient) and $3,508/female patient (average $65,285/patient) within two years of injury; 37 pre-injury factors were significantly associated with increased direct medical costs. Patients who first received care for their TBI in acute care (18.1%) incurred a median cost of $25,081/male patient (average $63,060/patient) and $30,277/female patient (average $65,285/patient) within two years of injury; 21 factors were significantly associated with increased direct medical costs. Among more prevalent factors, those associated with increased medical cost by at least 50% included mental health disorders, substance abuse, disorders or medical conditions frequently observed among the elderly, cardiovascular disorders, stroke and emergencies involving the brain, metabolic disorders and abdominal symptoms, conditions and symptoms of abdomen and pelvis, genitourinary disorders and disorders of prostate, and pulmonary abdominal and other emergencies.

**Conclusions:**

Direct medical costs two years post-TBI differed significantly between patients with and without adverse pre-existing health status. Interdisciplinary teams to promote early identification of pre-existing health conditions and appropriate management and integration of these conditions in TBI care across the continuum of healthcare may be opportunities to reduce direct medical costs post-injury.

## Introduction

Traumatic brain injury (TBI), defined as “an alteration in brain function, or other evidence of brain pathology, caused by an external force” [1], is a global public health concern. It was estimated that more than 50 million TBIs occur worldwide every year [2, 3], costing the global economy approximately $400 billion USD annually [2]. Indeed, in 2010 in the United States, 318,011 TBI-related admissions resulted in an estimated total cost of $21.4 billion USD or an average of $67,224 USD per visit; this cost estimate represented an increase of more than 200% from 2006 [4]. In Canada, between 2004 and 2007, the annual direct medical cost of patients hospitalized with a TBI was approximately $120.7 million CAD or $32,132 CAD per patient. Importantly, it has been demonstrated that patients with TBI continue to incur medical costs up to three years post injury in post-acute care (e.g., rehabilitation and community care) settings [5].

There is currently limited information on the impact of comorbidities on direct medical costs among survivors of TBI of all injury severities and across the lifespan. Comorbidities are particularly relevant because long-term health conditions are common [6, 7]. Concurrently, existing research has consistently concluded that the presence of more comorbid health conditions is associated with higher costs [4, 8]. However, the comorbidities captured in these studies were primarily based on self-reports or included only a select number of health conditions based on comorbidity indices that were neither developed nor validated for the TBI population [4, 8, 9]. Furthermore, even though it is well-established that the health experience and interactions with the health system differ between males and females with TBI [10–14], there is still a paucity of sex-stratified data to inform healthcare planning that is sensitive to the needs of male and female patients, specifically. Finally, as the world’s population ages, the demographics of individuals with TBI are likewise expected to increasingly include more older adults who are expected to accumulate a variety of short- and long-term health conditions that will impact health service use. As such, it is crucial to understand how pre-existing health status, defined as comorbid health conditions and personal, social, and environmental factors, affects direct medical cost after TBI to inform cost-effective healthcare planning and resource allocation at the health system level.

The objective of this population-based sex-stratified study was to understand how health status five-years preceding the TBI event affects direct medical costs two years post-injury in Ontario, Canada, home to 39% of the Canadian population [15]. Additionally, this study aimed to address existing gaps in the literature on comorbidities by identifying all health conditions and related health problems seen in the emergency department (ED) or acute care settings prior to the TBI event using data mining methodologies that categorized health status into 43 factors internally validated for the TBI population [16]. Findings from this paper provide the foundation to inform future research and practice opportunities to direct healthcare services to reduce the healthcare burden of patients with TBI on the health system.

## Materials and methods

This study was approved by research ethics boards at KITE-Toronto Rehab, University Health Network and at the University of Toronto. Written informed consent was not obtained from patients because this study accessed de-identified health administrative data.

### Sample

Patients age 19 years or older in the ED or acute care in the province of Ontario in Canada between April 1, 2007 and March 31, 2014 with a TBI diagnosis code were identified. TBI was defined using International Classification of Diseases Version 10 (ICD-10) codes: S02.0, S02.1, S02.3, S02.7, S02.8, S02.9, S04.0, S07.1, and S06; these codes have been used consistently to identify TBI in health administrative data and reviews of the literature have identified an association between TBI and the conditions described by these codes [5, 16–20]. For inclusion in the final sample, their TBI event must be the most responsible diagnosis (MRDx), defined as “the diagnosis or condition that can be described as being most responsible for the patient’s stay in a facility” (e.g., greatest portion of the length of stay or greatest use of resource) [21, 22]. The first healthcare encounter for a TBI (i.e., a health record in the ED or acute care with a TBI ICD-10 diagnosis code as the MRDx) during the study period was considered the first TBI event for this study. All patients who survive their healthcare encounter for two years were followed to determine their direct medical costs two years post-injury.

#### Data source

Population-based health administrative data were used. Data on ED visits were obtained from the Canadian Institute of Health Information National Ambulatory Care Reporting System (NACRS) and data on acute care visits were obtained from the Discharge Abstract Database (DAD). The NACRS contains demographic and clinical information on all ED visits in the province of Ontario; data quality reports on the NACRS indicate high agreement rates for the patients’ main problem [21]. The DAD contains demographic, clinical, discharge, transfer, and death information on all acute care hospitals in Ontario [21]; data quality on the MRDx variable in the DAD indicate that agreement for many diagnoses are good to excellent, with substantial agreement for the diagnosis code for intracranial injury (ICD-10 code S06) [23]. Data collection of the NACRS and DAD are mandatory in Ontario and as such, this study captured all patients in the province of Ontario who visited the ED or acute care setting during the study period.

### Variables

The outcome of interest was the total direct medical costs for all publicly funded health service utilization within two years of ED or acute care discharge, adjusted to the 2016 Canadian dollar. To determine this direct medical cost, a unit cost or price per service utilized during the encounter was identified for each patient [24]: costs associated with short episodes (acute care, ED, and same day surgery/outpatient, and inpatient rehabilitation) were calculated using their resource intensity weights (i.e., how much resources were used during each encounter) and the cost per weighted case [24]; costs associated with longer term episodes (complex continuing care/long-term care, mental health beds) were calculated based on weighted days; and costs for visits/claims (physician services, homecare) were identified directly from recorded information in the datasets [24]. Healthcare specific Consumer Price Index (CPI) reported by Statistics Canada and the corresponding inflation rate (percent change in CPI) were used to standardize costs to the year 2016. Specifically, costs associated with physician visits and homecare services were multiplied by the corresponding inflation rate for the CPI for healthcare services and costs associated with ED, acute care, same day surgery, inpatient rehabilitation, complex continuing care/long-term care, and mental health beds were multiplied by the corresponding inflation rate for the CPI for healthcare [24]. This methodology has been used by researchers in Ontario, Canada, to derive direct economic costs [5, 20, 24].

Demographic, injury-related, ED and/or acute care-related, and pre-injury health status variables were identified to describe the sample. Demographic variables included: (a) age at ED or acute care discharge; (b) rural residence (yes vs. no), determined based on the individual postal codes designated as being rural by the Canadian Postal Service; and (c) income quintile, defined as the relative household income adjusted for household size and community (Q1 = lowest, Q5 = highest).

Injury-related variables included: (a) injury severity, measured using the Abbreviated Injury Severity (AIS) and categorized as mild (1–2), moderate (3), severe (4+) and unspecified (i.e., diagnosis code was not specific enough to determine severity of injury) [25]; (b) mechanism of injury, categorized as falls, motor vehicle collision, struck by/against an object, and other [26]; and (c) sports-related (yes vs. no) [27].

ED and/or acute care-related variables included: (a) length of stay, defined as the number of hours (ED) or days (acute care) between admission and discharge; (b) special care days, defined as the number of days in intensive care unit (acute care only); and (c) alternate level of care days, defined as days in which patients are still occupying an acute care bed but their acute care treatment has finished (acute care only) [28].

Pre-injury health status was defined as comorbidities and personal, social, and environmental circumstances present between five years and 31 days (inclusive) preceding the TBI event. This time period was determined based on a histogram of all patients’ ED or acute care visit prior to and post-TBI, which revealed a peak of healthcare utilization 30-days before and after the TBI event. Healthcare utilization during this 60-day window was therefore determined to be TBI-related and healthcare utilization 31-days prior to the TBI event was considered pre-injury [16]. Therefore, all comorbidities and personal, social, and environmental circumstances present in patients’ ED and acute care records between five years and 31-days (inclusive) preceding the TBI event were identified and categorized into 43 factors using ICD-10 codes based on a data mining study on pre-injury health status among patients with TBI in Ontario [16]. These factors were established using multiple testing methods that identified significant differences in the frequency and odds of having a specified ICD-10 code for patients with TBI compared to a cohort of patients without TBI, matched on age, sex, rurality of residence, and income quintile [16]. Factor analyses were subsequently conducted to categorize these ICD-10 codes into 43 factors. Some ICD-10 codes were represented in multiple factors from this factor analysis, which may represent shared pathophysiological mechanisms of different disorders. These factors were internally validated by splitting the dataset into training, validation, and testing datasets. S1 Table lists the ICD-10 codes that are captured in the 43 factors and additional details on the methodology to establish this pre-injury timeframe and 43 factors are available in peer-reviewed manuscript on data mining to understand health status preceding TBI [16].

### Analyses

Sex-specific multivariable linear regressions were conducted for each of the 43 factors, controlling for age, income quintile (referent group = Q1), and rurality of residence (referent group = urban), with the log of the total direct medical costs as the outcome variable. The log transformation on the cost outcome variable was conducted because the distribution was highly skewed. All analyses were stratified by location of the first healthcare encounter (ED vs. acute care) during the study period for a TBI. This was also used as a proxy for injury severity because 75.2% of male and 67.0% of female patients in acute care had a severe TBI while 11.9% of male and 7.7% of female patients had a severe TBI in the ED. Bonferroni correction was applied to account for multiple testing and Bonferroni-adjusted confidence intervals were calculated. All graphs and modeling were conducted using SAS v9.4.

## Results

Between April 1, 2007 and March 31, 2014, there were 55,669 patients age 19 years or older (52.3% males; N = 29,139) with an ED or acute care visit for a TBI. The ED was the location of the first healthcare encounter during the study period for 81.9% of patients (N = 45,585) and the sex distribution of patients in the ED was almost equal (49.9% males; N = 22,770). The remaining patients (18.1%; N = 10,084) had their first healthcare encounter for their TBI in the acute care setting, of whom 63.2% (N = 6,359) were males. Table 1 presents the demographic, injury-related, and ED and/or acute care-related characteristics for each patient by age and first healthcare encounter location.

**Table 1:** Profile of patients in the emergency department or acute care for a TBI in Ontario, Canada, 2007/08–2013/14 (inclusive), by sample characteristics, sex, and location of first TBI healthcare encounter during the study period.

	Location of Encounter: Emergency Department N = 45,585 (81.9%)		Location of Encounter: Acute Care N = 10,084 (18.1%)	
	Male Patients N = 22,770	Female Patients N = 22,815	Male Patients N = 6,369	Female Patients N = 3,715
**Demographic**				
Age				
Mean ± SD	41.9 ± 18.3	44.8 ± 19.2	56.7 ± 20.6	64.7 ± 20.2
Median (IQR)	39.0 (26.0–54.0)	43.0 (28.0–58.0)	59.00 (40.0–74.0)	69.00 (52.0–81.0)
Rural Residence (Yes)	3,053 (13.4%)	3,098 (13.6%)	894 (14.0%)	427 (11.5%)
Income Quintile				
1 (Lowest)	4,470 (19.6%)	4,416 (19.4%)	1,329 (20.9%)	778 (20.9%)
2	4,612 (20.3%)	4,502 (19.7%)	1,315 (20.7%)	735 (19.8%)
3	4,477 (19.7%)	4,498 (19.7%)	1,249 (19.6%)	689 (18.6%)
4	4,641 (20.3%)	4,820 (21.1%)	1,261 (19.8%)	742 (19.9%)
5 (Highest)	4,570 (20.1%)	4,579 (20.1%)	1,215 (19.1%)	771 (20.8%)
**Injury-Related**				
Injury Severity (AIS)				
Unspecified	13,256 (58.2%)	15,822 (69.4%)	927 (14.6%)	748 (20.1%)
Mild	5,400 (23.7%)	4,626 (20.3%)	349 (5.5%)	264 (7.1%)
Moderate	1,404 (6.2%)	611 (2.7%)	303 (4.7%)	215 (5.8%)
Severe	2,710 (11.9%)	1,756 (7.7%)	4,790 (75.2%)	2,488 (67.0%)
Mechanism of Injury				
Fall	8,613 (37.8%)	11,721 (51.4%)	3,512 (55.1%)	2,398 (64.6%)
Motor Vehicle Collision	2,685 (11.8%)	2,631 (11.5%)	1,060 (16.6%)	733 (19.7%)
Struck By/Against	8,427 (37.0%)	6,160 (27.0%)	798 (12.5%)	166 (4.5%)
Other	3,033 (13.3%)	2,297 (10.1%)	992 (15.6%)	408 (10.9%)
Unknown	12 (0.05%)	6 (0.03%)	7 (0.1%)	10 (0.3%)
Sports-Related Injury (Yes)	4,904 (21.5%)	3,180 (13.9%)	512 (8.0%)	190 (5.1%)
**Clinical**				
Fiscal Year of Discharge				
2007/08	2,976 (13.1%)	2,378 (10.4%)	855 (13.4%)	491 (13.2%)
2008/09	2,842 (12.5%)	2,573 (11.3%)	842 (13.2%)	425 (11.4%)
2009/10	2,947 (12.9%)	2,829 (12.4%)	869 (13.6%)	519 (14.0%)
2010/11	3,114 (13.7%)	2,967 (13.0%)	888 (13.9%)	508 (13.7%)
2011/12	3,402 (14.9%)	3,341 (14.6%)	943 (14.8%)	553 (14.9%)
2012/13	3,545 (15.6%)	3,853 (16.9%)	972 (15.3%)	603 (16.2%)
2013/14	3,944 (17.3%)	4,874 (21.4%)	1,000 (15.7%)	616 (16.6%)
LOS (Days)				
Mean ± SD			9.8 ± 16.1	9.1 ± 14.0
Median (IQR)	-	-	5.0 (2.0–11.0)	5.0 (2.0–11.0)
LOS (Hours)				
Mean ± SD	4.9 ± 5.7	4.4 ± 5.8		
Median (IQR)	3.4 (2.0–5.0)	3.1 (1.8–4.9)	-	-
Special Care Days (Yes)	-	-	2,601 (40.8%)	1,224 (32.9%)
ALC Days				
Yes			942 (14.8%)	681 (18.3%)
Mean ± SD	-	-	18.5 ± 37.3	16.5 ± 34.0
Median (IQR)			8.0 (4.0–19.0)	7.0 (3.0–16.0)

**AIS:** Abbreviated Injury Severity: **ALC:** Alternate Level of Care; **IQR:** Interquartile Range; **LOS:** Length of Stay; **SD:** Standard Deviation.

The total direct medical cost of publicly funded health service utilization within two years of discharge was $1.21 billion CAD ($682.7 million among male and $524.8 million among female patients). The median cost per male patient over two years was $4,283 ($1,366 - $16,078) and among female patients, $4,578 ($1,914 - $13,249). During the study period, the number of male and female patients with TBI increased by 20.1% and 91.1%, respectively, but the median cost per patient decreased from $4,271 ($1,351 - $16,406) in 2007/08 to $3,966 ($1,357 - $15,463) in 2013/14 among male patients and from $4,911 ($1,986 - $15,497) in 2007/08 to $3,994 ($1,794 - $10,729) in 2013/14 among female patients. The 18.1% of patients who had their first healthcare encounter in the acute care accounted for 53.3% of the total direct medical cost over the two-year follow-up period. Table 2 presents the total direct medical cost of publicly funded healthcare utilization within two years of acute care discharge by location of first healthcare encounter.

**Table 2:** Total direct medical costs of publicly funded healthcare utilization, adjusted to the 2016 Canadian Dollar, within two years of emergency department or acute care discharge in Ontario, Canada, 2007/08–2013/14, by sex and location of first TBI encounter during the study period.

	Number of Unique Patients (Percent)	Cumulative Cost $CAD	Average Cost per Individual ± Standard Deviation	Median Cost per individual (Interquartile Range)
**Total**				
All	55,669 (100.0%)	$1,207,464,734	$21,690 ± 56,077	$4,446 ($1,634 - $14,675)
Location of First Healthcare Encounter				
Emergency Department	45,585 (81.9%)	$563,301,851	$12,357 ± $36,265	$3,026 ($1,364 - $8,414)
Acute Care	10,084 (18.1%)	$644,162,883	$63,880 ± $96,134	$26,779 ($12,361 - $78,222)
Fiscal Year of Discharge				
2007/08	6,701 (12.0%)	$151,359,866	$22,588 ± $55,308	$4,623 ($1,643 - $15,981)
2008/09	6,685 (12.0%)	$155,751,019	$23,299 ± $58,927	$4,772 ($1,677 - $15,734)
2009/10	7,168 (12.9%)	$160,333,864	$22,368 ± $56,366	$4,965 ($1,687 - $16,176)
2010/11	7,480 (13.4%)	$161,769,266	$21,627 ± $53,944	$4,561 ($1,676 - $14,975)
2011/12	8,239 (14.8%)	$184,089,418	$22,344 ± $60,377	$4,602 ($1,605 - $14,745)
2012/13	8,971 (16.1%)	$187,847,588	$20,939 ± $55,624	$4,227 ($1,600 - $13,822)
2013/14	10,425 (18.7%)	$206,313,713	$19,790 ± $52,709	$3,983 ($1,602 - $12,481)
**Male Patients**				
All	29,139 (100.0%)	$682,664,415	$23,428 ± $60,369	$4,283 ($1,366 - $16,078)
Location of First Healthcare Encounter				
Emergency Department	22,770 (78.1%)	$281,034,589	$12,342 ± $37,613	$2,492 ($1,095 - $7,864)
Acute Care	6,369 (21.9%)	$401,629,826	$63,060 ± $98,015	$25,081 ($11,961 - $74,377)
Fiscal Year of Discharge				
2007/08	3,831 (13.1%)	$89,142,829	$23,269 ± $57,861	$4,271 ($1,351 - $16,406)
2008/09	3,684 (12.6%)	$88,333,040	$23,977 ± $59,420	$4,611 ($1,367 - $17,063)
2009/10	3,817 (13.1%)	$91,872,478	$24,069 ± $62,801	$4,634 ($1,393 - $16,835)
2010/11	4,004 (13.7%)	$91,006,117	$22,729 ± $56,782	$4,454 ($1,459 - $16,331)
2011/12	4,346 (14.9%)	$105,017,395	$24,164 ± $66,491	$4,260 ($1,285 - $15,366)
2012/13	4,517 (15.5%)	$104,991,274	$23,244 ± $61,211	$4,062 ($1,348 - $15,301)
2013/14	4,940 (17.0%)	$112,301,282	$22,733 ± $57,392	$3,966 ($1,357 - $15,463)
**Female Patients**				
All	26,530 (100.0%)	$524,800,319	$19,781 ± $50,881	$4,578 ($1,914 - $13,249)
Location of First Healthcare Encounter				
Emergency Department	22,815 (86.0%)	$282,267,262	$12,372 ± $34,869	$3,508 ($1,698 - $8,825)
Acute Care	3,715 (14.0%)	$242,533,057	$65,285 ± $92,818	$30,277 ($13,260 - $82,619)
Fiscal Year of Discharge				
2007/08	2,870 (10.8%)	$62,217,037	$21,678 ± $51,702	$4,911 ($1,986 - $15,497)
2008/09	3,001 (11.3%)	$67,417,979	$22,465 ± $58,316	$4,947 ($1,994 - $14,383)
2009/10	3,351 (12.6%)	$68,461,386	$20,430 ± $47,931	$5,260 ($1,990 - $15,364)
2010/11	3,476 (13.1%)	$70,763,149	$20,358 ± $50,456	$4,606 ($1,953 - $13,898)
2011/12	3,893 (14.7%)	$79,072,023	$20,311 ± $52,654	$4,749 ($1,944 - $14,098)
2012/13	4,454 (16.8%)	$82,856,314	$18,603 ± $49,214	$4,315 ($1,834 - $12,575)
2013/14	5,485 (20.7%)	$94,012,431	$17,140 ± $47,952	$3,994 ($1,794 - $10,729)

**Fig 1:**
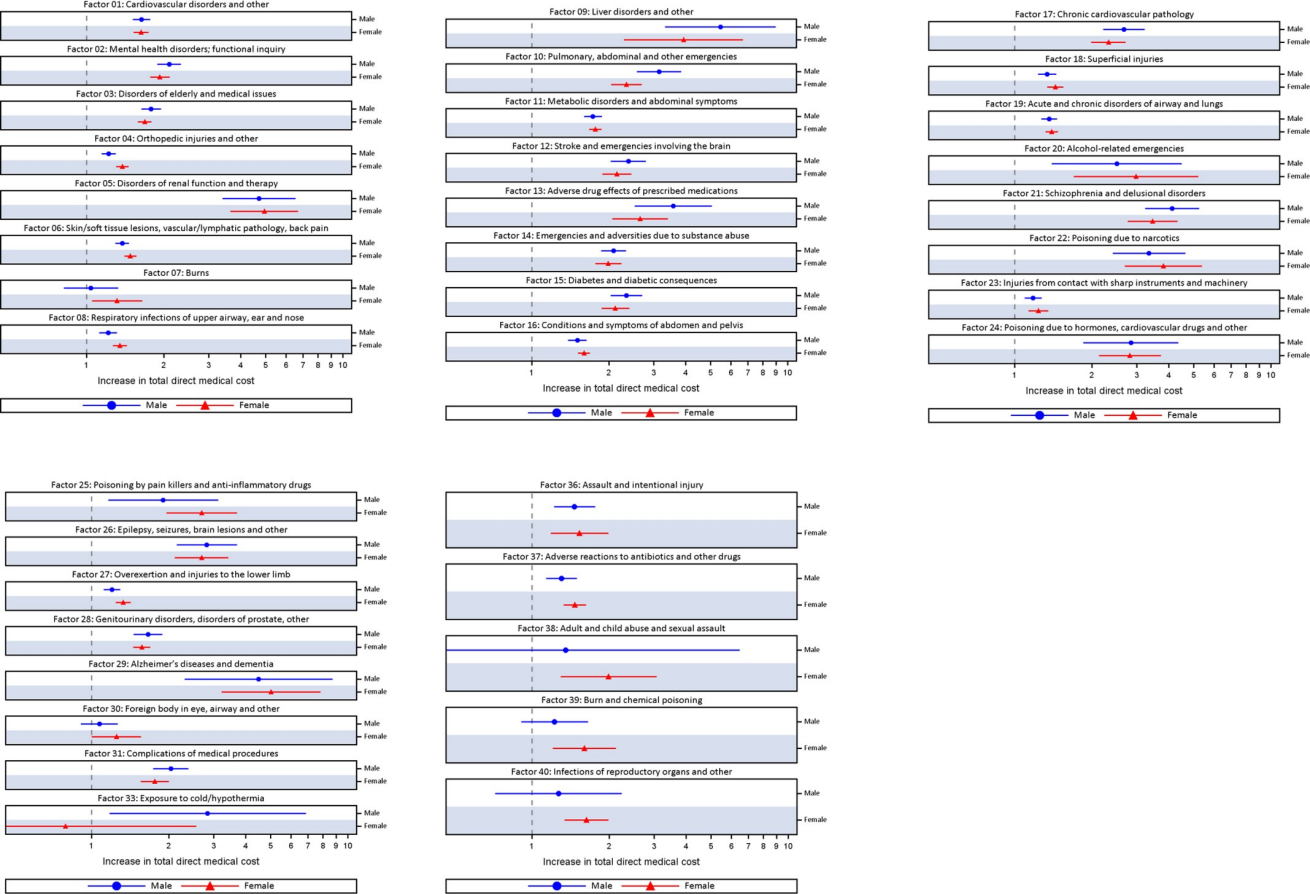
Bonferroni adjusted multivariable linear regression of pre-injury health status significantly associated with direct medical cost among patients in the emergency department for a TBI in Ontario, Canada, between 2007/08 and 2013/14, by sex.

Multivariable linear regressions among patients who first received care for their TBI in the ED setting identified 37 pre-injury health status factors that were significantly associated with increased direct medical cost. The direct medical costs of patients with the following more prevalent factors (i.e., present in more than 5% of the TBI population) were significantly increased compared to patients without these factors (factor number, % increase in cost): cardiovascular disorders (Factor 1; males 63.8%; females 63.2%), mental health disorders (Factor 2; males: 110%; females 93.3%), disorders or medical issues frequently observed among the elderly (Factor 3; males: 78.7%; females 68.4%), orthopedic injuries (Factor 4; males: 22.0%; females 37.9%), skin/soft tissue lesions, vascular/lymphatic pathology, back pain (Factor 6: males: 37.8%; females: 48.3%), respiratory infections of upper airway, ear and nose (Factor 8; males: 21.4%; females 34.7%), metabolic disorders (Factor 11; males: 73.4%; females: 77.1%), conditions and symptoms of abdomen and pelvis (Factor 16; males: 50.6%; females: 59.9%), superficial injuries (Factor 18; males: 33.9%; females: 47.1%), respiratory diseases (Factor 19; males: 36.5%; females: 39.5%), injuries from contact with sharp instruments and machinery (Factor 23; males: 18.0%; females: 32.7%), overexertion and injuries to the lower limb (Factor 27; males: 20.1%; females: 32.7%), and genitourinary disorders and disorders of prostate (Factor 28: males: 73.4%; females 77.1%). Fig 1 presents the multivariable linear regression models of pre-injury health status significantly associated with increased direct medical cost among patients in the ED for a TBI, including those not listed above (i.e., factors present in <5% of the TBI population). Table 3 presents the prevalence of pre-injury health status prior to the TBI and the multivariable linear regression models for direct medical cost among males and females in the ED.

**Table 3:** Prevalence of pre-injury health status and multivariable linear regression models for direct medical cost among patients who first received TBI care in the emergency department.

	Male Patients				Female Patients			
Factor Description	N (%)	Increase in Cost	Bonferroni Confidence Interval	p-value	N (%)	Increase in Cost	Bonferroni Confidence Interval	p-value
Factor 01: Cardiology—Cardiovascular disorders and other	3,313 (14.6%)	1.638	(1.513–1.773)	<0.0001*	3,860 (16.9%)	1.632	(1.528–1.744)	<0.0001*
Factor 02: Psychiatry—Mental health disorders; functional inquiry	1,601 (7.0%)	2.102	(1.890–2.338)	<0.0001*	1,972 (8.6%)	1.933	(1.771–2.108)	<0.0001*
Factor 03: Geriatrics—Disorders of elderly and medical issues	2,625 (11.5%)	1.787	(1.640–1.948)	<0.0001*	4,715 (20.7%)	1.684	(1.586–1.789)	<0.0001*
Factor 04: Trauma—Orthopedic injuries and other	6,146 (27.0%)	1.22	(1.146–1.299)	<0.0001*	5,408 (23.7%)	1.379	(1.302–1.461)	<0.0001*
Factor 05: Nephrology—Disorders of renal function and therapy	160 (0.7%)	4.701	(3.384–6.531)	<0.0001*	149 (0.7%)	4.933	(3.636–6.692)	<0.0001*
Factor 06: Dermatology osteopathy/orthopedy—Skin/soft tissue lesions, vascular/lymphatic pathology, back pain	5,989 (26.3%)	1.378	(1.295–1.467)	<0.0001*	6,603 (28.9%)	1.483	(1.405–1.566)	<0.0001*
Factor 07: Environmental exposures—Burns	291 (1.3%)	1.04	(0.814–1.328)	0.6031	273 (1.2%)	1.315	(1.049–1.649)	<0.0001*
Factor 08: Otolaryngology—Respiratory infections of upper airway, ear and nose	3,189 (14.0%)	1.214	(1.121–1.315)	<0.0001*	4,504 (19.7%)	1.347	(1.265–1.435)	<0.0001*
Factor 09: Gastroenterology—Liver disorders and other	69 (0.3%)	5.46	(3.319–8.982)	<0.0001*	48 (0.2%)	3.916	(2.291–6.692)	<0.0001*
Factor 10: Emergency medicine—Pulmonary, abdominal and other emergencies	445 (2.0%)	3.141	(2.576–3.831)	<0.0001*	738 (3.2%)	2.346	(2.042–2.694)	<0.0001*
Factor 11: Gastroenterology—Metabolic disorders and abdominal symptoms	3,167 (13.9%)	1.734	(1.602–1.876)	<0.0001*	5,503 (24.1%)	1.771	(1.674–1.874)	<0.0001*
Factor 12: Neurology—Stroke and emergencies involving the brain	752 (3.3%)	2.383	(2.036–2.788)	<0.0001*	884 (3.9%)	2.149	(1.886–2.448)	<0.0001*
Factor 13: Pharmacology emergencies—Adverse drug effects of prescribed medications	142 (0.6%)	3.574	(2.526–5.057)	<0.0001*	220 (1.0%)	2.648	(2.060–3.403)	<0.0001*
Factor 14: Toxicology—Emergencies and adversities due to substance abuse	1,452 (6.4%)	2.088	(1.868–2.335)	<0.0001*	1,044 (4.6%)	1.99	(1.769–2.237)	<0.0001*
Factor 15: Endocrinology—Diabetes and diabetic consequences	931 (4.1%)	2.343	(2.035–2.698)	<0.0001*	967 (4.2%)	2.121	(1.874–2.400)	<0.0001*
Factor 16: Gastroenterology and obstetrics—Conditions and symptoms of abdomen and pelvis	2,842 (12.5%)	1.506	(1.387–1.635)	<0.0001*	6,740 (29.5%)	1.599	(1.515–1.687)	<0.0001*
Factor 17: Cardiology—Chronic cardiovascular pathology	519 (2.3%)	2.674	(2.219–3.223)	<0.0001*	599 (2.6%)	2.323	(1.989–2.714)	<0.0001*
Factor 18: Trauma—Superficial injuries	2,852 (12.5%)	1.339	(1.232–1.454)	<0.0001*	3,098 (13.6%)	1.44	(1.340–1.547)	<0.0001*
Factor 19: Infectious diseases and respirology—Acute and chronic disorders of airway and lungs	4,211 (18.5%)	1.365	(1.272–1.465)	<0.0001*	5,679 (24.9%)	1.395	(1.318–1.476)	<0.0001*
Factor 20: Toxicology—Alcohol-related emergencies	50 (0.2%)	2.503	(1.394–4.492)	<0.0001*	44 (0.2%)	2.978	(1.701–5.215)	<0.0001*
Factor 21: Psychiatry—Schizophrenia and delusional disorders	289 (1.3%)	4.111	(3.224–5.242)	<0.0001*	275 (1.2%)	3.457	(2.763–4.325)	<0.0001*
Factor 22: Pharmacology emergencies—Poisoning due to narcotics	161 (0.7%)	3.341	(2.411–4.629)	<0.0001*	114 (0.5%)	3.805	(2.687–5.389)	<0.0001*
Factor 23: Trauma—Injuries from contact with sharp instruments and machinery	3,491 (15.3%)	1.18	(1.093–1.274)	<0.0001*	1,941 (8.5%)	1.237	(1.132–1.352)	<0.0001*
Factor 24: Pharmacology emergencies—Poisoning due to hormones, cardiovascular drugs and other	94 (0.4%)	2.842	(1.855–4.355)	<0.0001*	181 (0.8%)	2.817	(2.137–3.714)	<0.0001*
Factor 25: Pharmacology emergencies—Poisoning by pain killers and anti-inflammatory drugs	70 (0.3%)	1.904	(1.161–3.123)	<0.0001*	139 (0.6%)	2.689	(1.961–3.688)	<0.0001*
Factor 26: Neurology—Epilepsy, seizures, brain lesions and other	233 (1.0%)	2.816	(2.147–3.695)	<0.0001*	238 (1.0%)	2.686	(2.110–3.418)	<0.0001*
Factor 27: Trauma—Overexertion and injuries to the lower limb	3,784 (16.6%)	1.201	(1.115–1.294)	<0.0001*	3,612 (15.8%)	1.327	(1.240–1.420)	<0.0001*
Factor 28: Nephrology—Genitourinary disorders, disorders of prostate, other	1,098 (4.8%)	1.66	(1.459–1.889)	<0.0001*	2,673 (11.7%)	1.571	(1.456–1.695)	<0.0001*
Factor 29: Neurology—Alzheimer’s diseases and dementia	39 (0.2%)	4.481	(2.306–8.707)	<0.0001*	71 (0.3%)	5.014	(3.220–7.808)	<0.0001*
Factor 30: Emergency medicine—Foreign body in eye, airway and other	647 (2.8%)	1.072	(0.909–1.265)	0.1692	282 (1.2%)	1.25	(1.001–1.562)	0.0011*
Factor 31: Emergency medicine—Complications of medical procedures	698 (3.1%)	2.037	(1.738–2.388)	<0.0001*	892 (3.9%)	1.766	(1.556–2.004)	<0.0001*
Factor 32: Environmental exposures—Exposure to heat and light	16 (0.1%)	1.206	(0.429–3.391)	0.5561	9 (0.04%)	1.315	(0.381–4.539)	0.4722
Factor 33: Environmental exposures—Exposure to cold/hypothermia	22 (0.1%)	2.838	(1.176–6.853)	0.0001*	10 (0.04%)	0.79	(0.244–2.559)	0.5154
Factor 34: Environmental exposures—Bee, wasp and hornet stings	115 (0.5%)	1.228	(0.834–1.808)	0.0842	125 (0.6%)	1.248	(0.894–1.742)	0.0308
Factor 35: Infectious diseases—Viral conjunctivitis	17 (0.1%)	0.747	(0.274–2.038)	0.3459	27 (0.1%)	1.13	(0.553–2.311)	0.5793
Factor 36: Trauma—Assault and intentional injury	511 (2.2%)	1.465	(1.218–1.763)	<0.0001*	209 (0.9%)	1.533	(1.185–1.985)	<0.0001*
Factor 37: Pharmacology emergencies—Adverse reactions to antibiotics and other drugs	940 (4.1%)	1.305	(1.137–1.498)	<0.0001*	1,412 (6.2%)	1.471	(1.328–1.628)	<0.0001*
Factor 38: Trauma—Adult and child abuse and sexual assault	7 (0.03%)	1.352	(0.283–6.451)	0.5309	75 (0.3%)	1.991	(1.296–3.060)	<0.0001*
Factor 39: Environmental exposures—Burn and chemical poisoning	191 (0.8%)	1.225	(0.907–1.655)	0.0282	173 (0.8%)	1.602	(1.207–2.127)	<0.0001*
Factor 40: Gynecology—Infections of reproductory organs and other	53 (0.2%)	1.269	(0.719–2.241)	0.1733	355 (1.6%)	1.631	(1.336–1.990)	<0.0001*
Factor 41: Pharmacology emergencies—Poisoning due to drugs acting on autonomic nervous system	< 6	3.349	(0.527–21.268)	0.0337	12 (0.1%)	1.001	(0.343–2.927)	0.9967
Factor 42: Environmental exposures—Toxic effect of gases, fumes and vapors	72 (0.3%)	1.051	(0.645–1.713)	0.7394	53 (0.2%)	1.662	(0.997–2.770)	0.0012
Factor 43: Environmental exposures—Exposure to electrical current	32 (0.1%)	0.977	(0.470–2.031)	0.9189	18 (0.08%)	1.147	(0.478–2.754)	0.6108

**Notes:** * indicates statistically significant after Bonferroni Adjustment.

**Fig 2:**
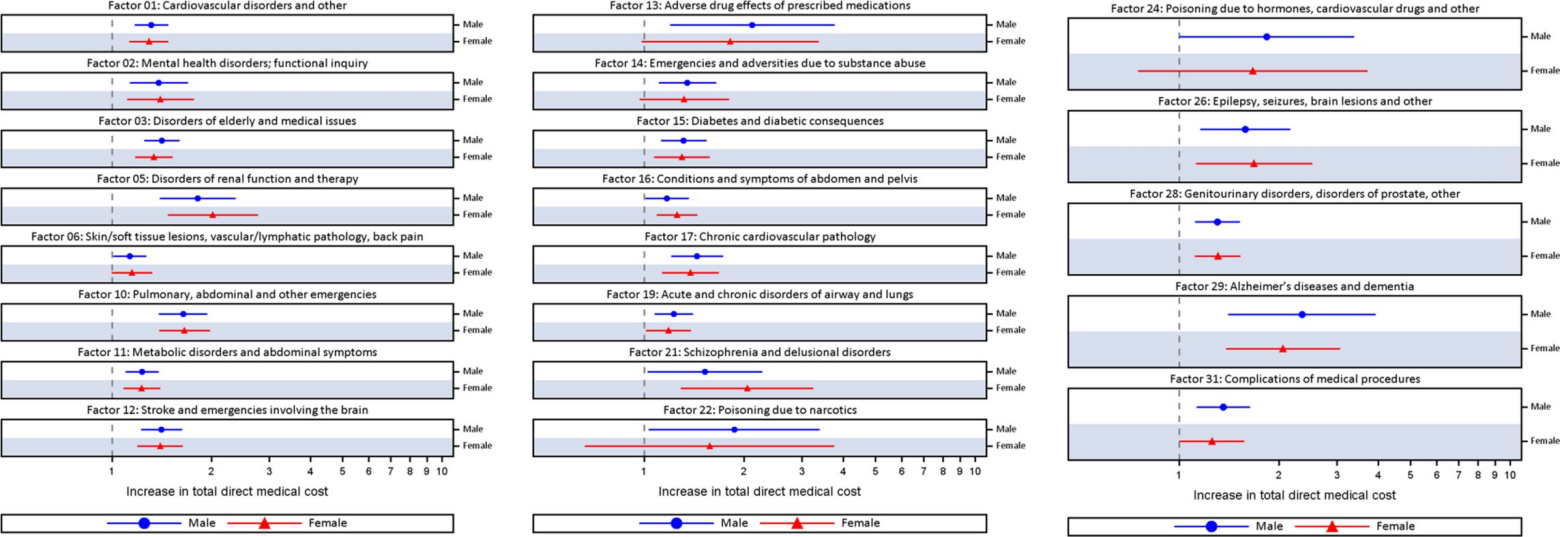
Bonferroni adjusted multivariable linear regression of pre-injury health status significantly associated with direct medical cost among patients in the acute care for a TBI in Ontario, Canada, between 2007/08 and 2013/14, by sex.

Multivariable linear regressions among individuals who first received TBI care in the acute care identified 21 pre-injury health status factors that were significantly associated with increased direct medical cost. The direct medical costs of patients with the following more prevalent factors (i.e., present in more than 5% of the TBI population) were significantly increased compared to patients without these factors (factor number, % increase in cost): cardiovascular disorders (Factor 1; males: 31.6%; females: 29.2% and Factor 17; males: 44.4%; females: 37.8%), mental health disorders (Factor 2; males: 38.6%; females: 40.2%), disorders or medical issues frequently observed among the elderly (Factor 3; males: 41.7%; females: 33.8%), pulmonary abdominal and other emergencies (Factor 10; males: 64.2%; females: 65.8%), metabolic disorders (Factor 11; males: 23.3%; females: 23.0%), stroke and emergencies involving the brain (Factor 12; males: 41.1% increase; females: 39.8% increase), diabetes (Factor 15; males: 31.6%; females: 30.0%), conditions and symptoms of abdomen and pelvis (Factor 16; males: 17.2%; females: 25.7%), respiratory diseases (Factor 19; males: 22.7%; females: 18.4%), genitourinary disorders and disorders of prostate (Factor 28; males: 30.3%; females: 30.7%), and complications of medical procedures (Factor 31; males: 35.9%; females: 25.5%). Five factors were significant only among males, which included skin/soft tissue lesions, vascular/lymphatic pathology, and back pain (Factor 6; 13.3%), substance abuse (Factor 14; 35.5%), and pharmacology emergencies (Factors 13, 22, and 24, which were of small sample sizes). Fig 2 presents the multivariable linear regression models of pre-injury health status significant associated with increased direct medical cost among patients in the acute care for a TBI. Table 4 presents the prevalence of pre-injury health status prior to the TBI and the multivariable linear regression models for direct medical cost among males and females in acute care.

**Table 4:** Prevalence of pre-injury health status and multivariable linear regression models for direct medical cost among patients who first received TBI care in the acute care setting.

	Male Patients				Female Patients			
Factor Description	N (%)	Increase in Cost	Bonferroni Confidence Interval	p-value	N (%)	Increase in Cost	Bonferroni Confidence Interval	p-value
Factor 01: Cardiology—Cardiovascular disorders and other	1,542 (24.2%)	1.316	(1.172–1.478)	<0.0001*	1,030 (27.7%)	1.292	(1.127–1.482)	<0.0001*
Factor 02: Psychiatry—Mental health disorders; functional inquiry	362 (5.7%)	1.386	(1.130–1.698)	<0.0001*	249 (6.7%)	1.402	(1.109–1.771)	<0.0001*
Factor 03: Geriatrics—Disorders of elderly and medical issues	1,277 (20.1%)	1.417	(1.254–1.601)	<0.0001*	1,147 (30.9%)	1.338	(1.174–1.526)	<0.0001*
Factor 04: Trauma—Orthopedic injuries and other	905 (14.2%)	1.134	(0.991–1.299)	0.0025	572 (15.4%)	1.172	(0.997–1.378)	0.0014
Factor 05: Nephrology—Disorders of renal function and therapy	210 (3.3%)	1.816	(1.394–2.365)	<0.0001*	131 (3.5%)	2.021	(1.473–2.773)	<0.0001*
Factor 06: Dermatology osteopathy/orthopedy—Skin/soft tissue lesions, vascular/lymphatic pathology, back pain	1,366 (21.5%)	1.133	(1.010–1.270)	0.0004*	822 (22.1%)	1.147	(0.996–1.322)	0.0016
Factor 07: Environmental exposures—Burns	40 (0.6%)	1.19	(0.658–2.154)	0.3408	14 (0.4%)	0.974	(0.375–2.530)	0.9277
Factor 08: Otolaryngology—Respiratory infections of upper airway, ear and nose	454 (7.1%)	1.107	(0.921–1.329)	0.0725	315 (8.5%)	1.155	(0.934–1.427)	0.0273
Factor 09: Gastroenterology—Liver disorders and other	73 (1.2%)	1.285	(0.827–1.996)	0.0643	44 (1.2%)	1.322	(0.770–2.270)	0.0936
Factor 10: Emergency medicine—Pulmonary, abdominal and other emergencies	551 (8.7%)	1.642	(1.385–1.945)	<0.0001*	471 (12.7%)	1.658	(1.387–1.981)	<0.0001*
Factor 11: Gastroenterology—Metabolic disorders and abdominal symptoms	1,549 (24.3%)	1.233	(1.100–1.382)	<0.0001*	1,173 (31.8%)	1.23	(1.081–1.400)	<0.0001*
Factor 12: Neurology—Stroke and emergencies involving the brain	865 (13.6%)	1.411	(1.223–1.629)	<0.0001*	678 (18.3%)	1.398	(1.193–1.637)	<0.0001*
Factor 13: Pharmacology emergencies—Adverse drug effects of prescribed medications	43 (0.7%)	2.122	(1.197–3.761)	<0.0001*	34 (0.9%)	1.817	(0.982–3.360)	0.0016
Factor 14: Toxicology—Emergencies and adversities due to substance abuse	380 (6.0%)	1.35	(1.106–1.647)	<0.0001*	137 (3.7%)	1.32	(0.966–1.804)	0.0039
Factor 15: Endocrinology—Diabetes and diabetic consequences	659 (10.4%)	1.316	(1.123–1.541)	<0.0001*	395 (10.6%)	1.3	(1.072–1.575)	<0.0001*
Factor 16: Gastroenterology and obstetrics—Conditions and symptoms of abdomen and pelvis	688 (10.8%)	1.172	(1.008–1.364)	0.0007*	825 (22.2%)	1.257	(1.092–1.446)	<0.0001*
Factor 17: Cardiology—Chronic cardiovascular pathology	487 (7.7%)	1.444	(1.205–1.731)	<0.0001*	373 (10.0%)	1.378	(1.130–1.681)	<0.0001*
Factor 18: Trauma—Superficial injuries	450 (7.1%)	1.035	(0.861–1.243)	0.5438	296 (8.0%)	1.201	(0.967–1.490)	0.006
Factor 19: Infectious diseases and respirology—Acute and chronic disorders of airway and lungs	921 (14.5%)	1.227	(1.073–1.403)	<0.0001*	628 (16.9%)	1.184	(1.013–1.385)	0.0004*
Factor 20: Toxicology—Alcohol-related emergencies	15 (0.2%)	1.701	(0.647–4.474)	0.0743	8 (0.2%)	1.806	(0.511–6.379)	0.1282
Factor 21: Psychiatry—Schizophrenia and delusional disorders	89 (1.4%)	1.525	(1.022–2.275)	0.0006*	61 (1.6%)	2.045	(1.289–3.244)	<0.0001*
Factor 22: Pharmacology emergencies—Poisoning due to narcotics	40 (0.6%)	1.87	(1.032–3.387)	0.0006*	17 (0.5%)	1.577	(0.663–3.752)	0.0879
Factor 23: Trauma—Injuries from contact with sharp instruments and machinery	593 (9.3%)	1.111	(0.944–1.307)	0.0362	181 (4.9%)	0.97	(0.739–1.275)	0.7199
Factor 24: Pharmacology emergencies—Poisoning due to hormones, cardiovascular drugs and other	38 (0.6%)	1.84	(1.002–3.379)	0.0011*	20 (0.5%)	1.668	(0.751–3.708)	0.0374
Factor 25: Pharmacology emergencies—Poisoning by pain killers and anti-inflammatory drugs	11 (0.2%)	1.613	(0.522–4.990)	0.1688	13 (0.4%)	2.123	(0.787–5.726)	0.0137
Factor 26: Neurology—Epilepsy, seizures, brain lesions and other	145 (2.3%)	1.584	(1.158–2.168)	<0.0001*	79 (2.1%)	1.684	(1.123–2.524)	<0.0001*
Factor 27: Trauma—Overexertion and injuries to the lower limb	496 (7.8%)	1.004	(0.841–1.199)	0.9391	278 (7.5%)	1.065	(0.852–1.331)	0.3604
Factor 28: Nephrology—Genitourinary disorders, disorders of prostate, other	673 (10.6%)	1.303	(1.114–1.523)	<0.0001*	619 (16.7%)	1.307	(1.117–1.530)	<0.0001*
Factor 29: Neurology—Alzheimer’s diseases and dementia	54 (0.9%)	2.351	(1.408–3.927)	<0.0001*	83 (2.2%)	2.057	(1.382–3.061)	<0.0001*
Factor 30: Emergency medicine—Foreign body in eye, airway and other	114 (1.8%)	1.158	(0.814–1.650)	0.1766	28 (0.8%)	1.033	(0.525–2.034)	0.8747
Factor 31: Emergency medicine—Complications of medical procedures	443 (7.0%)	1.359	(1.128–1.638)	<0.0001*	272 (7.3%)	1.255	(1.002–1.572)	0.0011*
Factor 32: Environmental exposures—Exposure to heat and light	< 6	3.707	(0.571–24.069)	0.0229	-			
Factor 33: Environmental exposures—Exposure to cold/hypothermia	< 6	1.07	(0.123–9.284)	0.9193	< 6	0.78	(0.022–27.667)	0.8215
Factor 34: Environmental exposures—Bee, wasp and hornet stings	24 (0.4%)	0.648	(0.301–1.391)	0.0649	9 (0.2%)	0.902	(0.275–2.961)	0.7773
Factor 35: Infectious diseases—Viral conjunctivitis	< 6	0.265	(0.019–3.735)	0.1032	< 6	0.725	(0.092–5.693)	0.6121
Factor 36: Trauma—Assault and intentional injury	78 (1.2%)	1.16	(0.757–1.777)	0.2593	33 (0.9%)	0.958	(0.514–1.787)	0.824
Factor 37: Pharmacology emergencies—Adverse reactions to antibiotics and other drugs	155 (2.4%)	1.054	(0.777–1.429)	0.575	103 (2.8%)	1.298	(0.909–1.854)	0.0174
Factor 38: Trauma—Adult and child abuse and sexual assault	-				< 6	0.913	(0.185–4.502)	0.8526
Factor 39: Environmental exposures—Burn and chemical poisoning	28 (0.4%)	1.207	(0.594–2.451)	0.389	8 (0.2%)	1.739	(0.492–6.150)	0.1545
Factor 40: Gynecology—Infections of reproductory organs and other	14 (0.2%)	1.005	(0.369–2.734)	0.9868	26 (0.7%)	1.638	(0.813–3.301)	0.0223
Factor 41: Pharmacology emergencies—Poisoning due to drugs acting on autonomic nervous system	< 6	3.121	(0.360–27.066)	0.087	< 6	2.905	(0.590–14.311)	0.0299
Factor 42: Environmental exposures—Toxic effect of gases, fumes and vapors	14 (0.2%)	0.887	(0.326–2.414)	0.6969	< 6	1.043	(0.084–12.981)	0.9564
Factor 43: Environmental exposures—Exposure to electrical current	< 6	1.077	(0.124–9.339)	0.9113	< 6	0.463	(0.037–5.766)	0.3214

**Notes:** * indicates statistically significant after Bonferroni Adjustment.

## Discussion

Between April 2007 and March 2014 in Ontario, Canada, 55,669 unique patients (almost 8,000 patients per year) with a TBI diagnostic code as the most responsible diagnosis received care in the ED or acute care settings. Approximately 4 in 5 patients first received care for their TBI in the ED setting and, within two years of their TBI, incurred a median cost of publicly insured services of $2,492 per patient among males and $3,508 among females. Patients who first received care for their TBI in an acute care setting incurred a median cost of $25,081 per patient within two years of their TBI among males and $30,277 among females. This population-based sex-stratified study demonstrated that direct medical costs post-injury was significantly affected by pre-existing health conditions. Among the 43 pre-injury factors explored in this study, 37 factors significantly increased direct medical costs among those who received care in the ED and 21 factors significantly increased direct medical costs among those who received care in the acute care setting. Among more prevalent factors, those that increased direct medical cost by at least 50% included mental health disorders and substance abuse (Factors 2 and 14), disorders or medical conditions frequently observed among the elderly (Factor 3), cardiovascular disorders (Factor 1), stroke and emergencies involving the brain (Factor 12), metabolic disorders and abdominal symptoms (Factor 11), conditions and symptoms of abdomen and pelvis (Factor 16), genitourinary disorders and disorders of prostate (Factor 28), and pulmonary abdominal and other emergencies (Factor 10). It is acknowledged that some of the less prevalent factors in this study, such as renal disorders (present in <1% of patients in ED and <4% of patients in acute care), had an even greater impact on direct medical costs than the factors listed above. Continued research on each significant pre-injury health condition among patients with TBI is encouraged. This includes exploring the types of healthcare services that are used post-injury, as well as the frequency of use, to inform healthcare planning that meets the needs of patients with TBI and to identify opportunities to reduce direct medical costs.

As stated above, of the 43 pre-injury health status examined in this study, the associations between direct medical cost and mental health disorders (Factor 2) and substance abuse (Factor 14) were among the greatest. This is not surprising because it is well established that a concurrent TBI and mental health or substance abuse diagnosis at the time of healthcare admission significantly predicts problematic health system level outcomes such as hospital readmissions and delayed discharges from acute care [19, 29]. These are significant policy concerns that are resource intensive and associated with increased healthcare cost and reduced quality of care. This study provided evidence that the existing health system is already treating individuals with prior mental health disorders or substance abuse, where these conditions present five-years prior to the TBI event increased direct medical cost by up to 110%. Unfortunately, to date, care for individuals with TBI and mental health and/or addictions remains fragmented with lack of appropriate services and support for these individuals across the continuum of care [30–32]. This need for integrated healthcare has been recognized globally and across many health conditions and settings [33–39] and is particularly important for survivors of TBI because sequalae from TBI, including but not limited to increased impulsivity, slowed thinking, and impaired reasoning, problem solving and social communication [40, 41], may lead to loss of stable housing, employment, and relationships [41, 42]. Therefore, integrated TBI and mental health/addictions care should be explored to reduce both the direct and indirect medical cost by reducing the likelihood of negative system level outcomes and improve quality of life and community re-integration.

The finding that disorders or medical conditions frequently observed among the elderly increased medical costs by up to 79% is of particular significance for the TBI population because recent research on TBI have noted changes in the epidemiological patterns of TBI such that there is an increase in older adults, particularly among females [2, 10]. Concurrently, the United Nations projected that by the year 2050, approximately one in six people in the world will be 65 years of age or older [43]. As such, the health systems worldwide may expect to see more older patients with TBI, particularly older female patients, and must be prepared to support this aging population through best practices or clinical guidelines specifically for the aging population. Furthermore, conditions captured in this factor include gait or mobility abnormalities, symptoms and signs related to cognitive functions and awareness, and falls from wheelchair, bed, and slipping, tripping and stumbling on the same level, suggesting that early intervention addressing cognitive and physical challenges as well as prevention of falls may be opportunities to address subsequent healthcare costs. Crucially, prevention of falls may also prevent the TBI event, as it is well-established, and supported by data from this study, that falls is the most common cause of TBI among older adults [2, 44].

Other pre-injury health conditions that were prevalent among patients with TBI included chronic health conditions that are increasingly common among adults overall [45, 46], suggesting that more adults may enter the health system with pre-existing health conditions that affect resource utilization. Among patients with TBI, chronic conditions may present added challenges and complexity to care at the time of injury as well as post-injury. As such, research exploring the potential impact of introducing interdisciplinary teams to support early identification and effective management of chronic health conditions during treatment is encouraged. In particular, research to identify gaps in care post-injury, particularly in the community setting, may inform discharge planning and post-injury care, which holds the potential to reduce the economic and personal costs of TBI.

Finally, sex differences were also observed between male and female patients’ direct medical costs within two years of discharge. Among patients who first received care in the ED setting, the proportion of males and females was approximately equal but the median direct medical cost among females was 41% higher than that of males. Similarly, even though 63% of patients who first received care in the acute care setting were males, the median direct medical cost among females was 21% higher than that of males. The higher median cost among females may reflect increased use of healthcare services with increasing age, as the median age of female patients in both the ED and acute care in this study was higher than males (43 vs. 39 years in ED and 69 vs. 59 years in acute care). It may also reflect sex/gender differences in the use of health services between males and females whereby women were found to be more likely to seek healthcare and medical attention compared to men [47–49]. Finally, it is noteworthy that the number of female patients with TBI increased by 91.1% during the study period while the number of male patients with TBI increased by 20.1%. The increase in female patients with TBI is consistent with recent literature [2, 10] and may be attributed to factors such as increasing awareness and reporting of TBI in activities (e.g., figure skating, cheerleading) and occupations (e.g., nursing, teachers) [50–52] that are more common among females. As such, in-depth sex-specific research into the types of health services used post-discharge is encouraged to better understand the sex-specific health services used post-injury to inform sex- and/or gender-sensitive healthcare planning.

### Strengths and limitations

A limitation of this study is that the direct medical costs reported in this study are underestimates of the true direct medical cost of TBI, as they do not capture patients who are undiagnosed or sustained a TBI but do not seek medical care in the ED or acute care. This study also excluded patients who do not survive two years post-injury because they may differ from those who survive at least two years post-injury in terms of healthcare utilization as well as pre-injury health status. As such, it is acknowledged that the direct medical costs of TBI is likely much higher than what was reported in this study. A second limitation is that this study conducted 43 sex-specific multivariable linear regressions, which do not account for multiple pre-injury health status. The objective of this study, however, was to understand how each pre-injury health status impact direct medical cost to provide the foundation to explore comorbidities and related health problems. As such, this is an appropriate and important first step to understand how comorbidities impact direct medical cost to support cost-effective healthcare planning. Data on sociodemographic variables known to impact outcomes after TBI (e.g., marital status, race/ethnicity) were unavailable in our data. Finally, pre-injury health status identified in this study only included those coded in the ED or hospitalization settings and thus, may miss comorbidities that are seen in primary care settings.

Despite these limitations, there are considerable strengths to this study, one of which is the use of a data mining approach to identify and categorize all ICD-10 codes present in emergency department and hospitalization health records of patients with TBI up to five-years prior to the TBI event into 43 factors internally validated for the TBI population [16]. This approach may be applied to other health conditions to understand the impact of comorbidities on diverse outcomes. A second strength is that there is mandatory reporting of ED and acute care visits in Ontario, Canada and as such, this study captured all adult patients in the ED or acute care for a TBI between April 1, 2007 and March 31, 2014. Unique de-identified patient IDs also enabled a longer look-back period of five-years prior to the TBI event and a two year follow-up period post-discharge to capture all of the patients’ interaction with the health system. Finally, all data in this study were stratified by sex, contributing to the growing literature on sex differences and similarities in the TBI population.

## Conclusion

This is the first population-based study, to the best of our knowledge, to demonstrate the financial burden of pre-existing health conditions on adult patients with TBI. Among patients who first received TBI care in the ED setting, 37 of 43 factors were significantly associated with increased direct medical costs and among patients who first received TBI care in the acute care setting, 21 of 43 factors were significantly associated with increased direct medical costs. Of particular relevance to the TBI population is that the direct medical costs of patients with pre-existing mental health disorders and substance abuse was increased by up to 110% compared to patients without these health conditions, supporting the need to explore integrated TBI and mental health/addictions care to promote early intervention and appropriate care across the continuum of healthcare. Furthermore, many of the significant factors identified in this study were chronic health conditions, suggesting that early intervention and management of chronic health conditions may be opportunities to reduce the financial burden of pre-injury health status after TBI. Finally, continued sex-stratified research on each factor that was found to be significantly associated with increased direct medical cost is encouraged to inform policies, prevention strategies, and interventions and supports that can address the needs of males and females with TBI.

## Supporting information

S1 Table International Classification of Diseases version 10 (ICD-10) codes captured in each pre-injury health status factor [Adapted with permission from Mollayeva et al. (2019). Data mining to understand health status preceding traumatic brain injury. Scientific Reports, 9: 5574]. (DOCX)
