# Supplementary material for: A population-based sex-stratified study to understand how health status preceding traumatic brain injury affects direct medical cost
Source: PLoS One. 2020 Oct 13;15(10):e0240208. doi: 10.1371/journal.pone.0240208 (PMC7553294; doi:10.1371/journal.pone.0240208)
Supplement: S1 Table — (DOCX) [file pone.0240208.s001.docx]

**Supplementary File**

**Title:** A population-based sex-stratified study to understand how health status preceding traumatic brain injury affects direct medical cost

**Supplementary Table 1.** International Classification of Diseases Version 10 (ICD-10) codes captured in each pre-injury health status factor [Adapted with permission from Mollayeva et al. (2019). Data mining to understand health status preceding traumatic brain injury. Scientific Reports, 9: 5574].

| **Factor Number** | **Factor Description and ICD-10 Codes** | |
| --- | --- | --- |
|  |  |  |
| 1 | **Cardiology - Cardiovascular disorders and other** | |
|  | I25 | Chronic ischemic heart disease |
|  | I21 | ST elevation (STEMI) and non-ST elevation (NSTEMI) myocardial infarction |
|  | R94 | Abnormal results of function studies |
|  | Z95 | Presence of cardiac and vascular implants and grafts |
|  | I20 | Angina pectoris |
|  | E78 | Disorders of lipoprotein metabolism and other lipidaemias |
|  | I24 | Other acute ischemic heart diseases |
|  | I10 | Essential (primary) hypertension |
|  | I50 | Heart failure |
|  | I48 | Atrial fibrillation and flutter |
|  | I35 | Nonrheumatic aortic valve disorders |
|  | R07 | Pain in throat and chest |
|  | E11 | Type 2 diabetes mellitus |
| 2 | **Psychiatry - Mental health disorders; functional inquiry** | |
|  | R45 | Symptoms and signs involving emotional state |
|  | F32 | Major depressive disorder, single episode |
|  | F43 | Reaction to severe stress, and adjustment disorders |
|  | F60 | Specific personality disorders |
|  | Z63 | Other problems related to primary support group, including family circumstances |
|  | F91 | Conduct disorders |
|  | Z91 | Personal risk factors, not elsewhere classified |
|  | X78 | Intentional self-harm by sharp object |
|  | F31 | Bipolar disorder |
|  | F41 | Other anxiety disorders |
|  | F33 | Major depressive disorder, recurrent |
|  | F34 | Persistent mood [affective] disorders |
|  | F90 | Attention-deficit hyperactivity disorders |
|  | X61 | Intentional self-poisoning by and exposure to antiepileptic, sedative-hypnotic, antiparkinsonism and psychotropic drugs, not elsewhere classified |
|  | F61 | Mixed and other personality disorders |
|  | X69 | Intentional self-poisoning by and exposure to other and unspecified chemicals and noxious substances |
|  | F39 | Unspecified mood [affective] disorder |
|  | Z59 | Problems related to housing and economic circumstances |
|  | F69 | Unspecified disorder of adult personality and behavior |
|  | X60 | Intentional self-poisoning by and exposure to nonopioid analgesics, antipyretics and antirheumatics |
|  | F19 | Other psychoactive substance related disorders |
|  | Z60 | Problems related to social environment |
|  | T43 | Poisoning by, adverse effect of and underdosing of psychotropic drugs, not elsewhere classified |
|  | F12 | Cannabis related disorders |
|  | Z56 | Problems related to employment and unemployment |
|  | Z65 | Problems related to other psychosocial circumstances |
|  | T65 | Toxic effect of other and unspecified substances |
| 3 | **Geriatrics - Disorders of elderly and medical issues** | |
|  | Z75 | Problems related to medical facilities and other health care |
|  | R29 | Other symptoms and signs involving the nervous and musculoskeletal systems |
|  | S72 | Fracture of femur |
|  | F03 | Unspecified dementia |
|  | R64 | Cachexia |
|  | G20 | Parkinson disease |
|  | F05 | Delirium, not induced by alcohol and other psychoactive substances |
|  | R41 | Other symptoms and signs involving cognitive functions and awareness |
|  | W19 | Unspecified fall |
|  | W05 | Fall from non-moving wheelchair, nonmotorized scooter and motorized mobility scooter |
|  | W01 | Fall on same level from slipping, tripping and stumbling |
|  | R26 | Abnormalities of gait and mobility |
|  | W18 | Other slipping, tripping and stumbling and falls |
|  | F02 | Dementia in other diseases classified elsewhere |
|  | M81 | Osteoporosis without current pathological fracture |
|  | Z50 | Care involving use of rehabilitation procedures |
|  | I10 | Essential (primary) hypertension |
|  | N39 | Other disorders of urinary system |
|  | Z74 | Problems related to care provider dependency |
|  | W06 | Fall from bed |
|  | E87 | Other disorders of fluid, electrolyte and acid-base balance |
|  | Z73 | Problems related to life management difficulty |
|  | R53 | Malaise and fatigue |
| 4 | **Trauma - Orthopedic injuries and other** | |
|  | S62 | Fracture at wrist and hand level |
|  | S69 | Other and unspecified injuries of wrist, hand and finger(s) |
|  | W22 | Striking against or struck by other objects |
|  | S60 | Superficial injury of wrist, hand and fingers |
|  | S63 | Dislocation and sprain of joints and ligaments at wrist and hand level |
|  | S52 | Fracture of forearm |
|  | W21 | Striking against or struck by sports equipment |
|  | Z47 | Orthopedic aftercare |
|  | W51 | Accidental striking against or bumped into by another person |
|  | W02 | Fall involving ice-skates, skis, roller-skates or skateboards |
|  | W23 | Caught, crushed, jammed or pinched in or between objects |
|  | S50 | Superficial injury of elbow and forearm |
|  | W19 | Unspecified fall |
|  | S59 | Other and unspecified injuries of elbow and forearm |
|  | V18 | Pedal cycle rider injured in noncollision transport accident |
|  | X59 | Exposure to unspecified factor |
|  | W18 | Other slipping, tripping and stumbling and falls |
|  | W01 | Fall on same level from slipping, tripping and stumbling |
|  | S90 | Superficial injury of ankle, foot and toes |
|  | X50 | Overexertion and strenuous or repetitive movements |
|  | W50 | Accidental hit, strike, kick, twist, bite or scratch by another person |
| 5 | **Nephrology - Disorders of renal function and therapy** | |
|  | N18 | Chronic kidney disease (CKD) |
|  | Z49 | Encounter for care involving renal dialysis |
|  | Z99 | Dependence on enabling machines and devices, not elsewhere classified |
|  | I12 | Hypertensive chronic kidney disease |
|  | N08 | Glomerular disorders in diseases classified elsewhere |
|  | N19 | Unspecified kidney failure |
|  | T82 | Complications of cardiac and vascular prosthetic devices, implants and grafts |
|  | N17 | Acute kidney failure |
| 6 | **Dermatology osteopathy/orthopedy - Skin/soft tissue lesions, vascular/lymphatic pathology, back pain** | |
|  | L03 | Cellulitis and acute lymphangitis |
|  | Z51 | Encounter for other aftercare |
|  | M79 | Other and unspecified soft tissue disorders, not elsewhere classified |
|  | L02 | Cutaneous abscess, furuncle and carbuncle |
|  | M25 | Other joint disorder, not elsewhere classified |
|  | M54 | Dorsalgia |
|  | R22 | Localized swelling, mass and lump of skin and subcutaneous tissue |
|  | L08 | Other local infections of skin and subcutaneous tissue |
|  | Z09 | Follow-up examination after treatment for conditions other than malignant neoplasms |
|  | I80 | Phlebitis and thrombophlebitis |
|  | R07 | Pain in throat and chest |
|  | Z48 | Encounter for other postprocedural aftercare |
|  | Z76 | Persons encountering health services in other circumstances |
|  | L97 | Non-pressure chronic ulcer of lower limb, not elsewhere classified |
| 7 | **Environmental exposures - Burns** | |
|  | T31 | Burns classified according to extent of body surface involved |
|  | T23 | Burn and corrosion of wrist and hand |
|  | X10 | Contact with hot drinks, food, fats and cooking oils |
|  | T22 | Burn and corrosion of shoulder and upper limb, except wrist and hand |
|  | X12 | Contact with other hot fluids |
|  | T21 | Burn and corrosion of trunk |
|  | T24 | Burn and corrosion of lower limb, except ankle and foot |
|  | X15 | Contact with hot household appliances |
|  | T20 | Burn and corrosion of head, face, and neck |
|  | T25 | Burn and corrosion of ankle and foot |
|  | X09 | Exposure to unspecified smoke, fire and flames |
|  | X19 | Contact with other heat and hot substances |
|  | X11 | Contact with hot tap-water |
|  | X08 | Exposure to other specified smoke, fire and flames |
| 8 | **Otolaryngology - Respiratory infections of upper airway, ear and nose** | |
|  | H66 | Suppurative and unspecified otitis media |
|  | J06 | Acute upper respiratory infections of multiple and unspecified sites |
|  | J02 | Acute pharyngitis |
|  | R50 | Fever of other and unknown origin |
|  | H92 | Otalgia and effusion of ear |
|  | B34 | Viral infection of unspecified site |
|  | R21 | Rash and other nonspecific skin eruption |
|  | R05 | Cough |
|  | J05 | Acute obstructive laryngitis [croup] and epiglottitis |
|  | J03 | Acute tonsillitis |
|  | H60 | Otitis externa |
|  | H10 | Conjunctivitis |
|  | J20 | Acute bronchitis |
| 9 | **Gastroenterology - Liver disorders and other** | |
|  | K74 | Fibrosis and cirrhosis of liver |
|  | K72 | Hepatic failure, not elsewhere classified |
|  | R18 | Ascites |
|  | I85 | Esophageal varices |
|  | K76 | Other diseases of liver |
|  | K70 | Alcoholic liver disease |
|  | B18 | Chronic viral hepatitis |
|  | D61 | Other aplastic anemias and other bone marrow failure syndromes |
| 10 | **Emergency medicine - Pulmonary, abdominal and other emergencies** | |
|  | A41 | Other sepsis |
|  | J17 | Pneumonia in diseases classified elsewhere |
|  | J96 | Respiratory failure, not elsewhere classified |
|  | R57 | Shock, not elsewhere classified |
|  | N17 | Acute kidney failure |
|  | B95 | Streptococcus, Staphylococcus, and Enterococcus as the cause of diseases classified elsewhere |
|  | B96 | Other bacterial agents as the cause of diseases classified elsewhere |
|  | E87 | Other disorders of fluid, electrolyte and acid-base balance |
|  | L89 | Pressure ulcer |
|  | F05 | Delirium, not induced by alcohol and other psychoactive substances |
|  | U82 | Resistance to betalactam antibiotics |
|  | J15 | Bacterial pneumonia, not elsewhere classified |
|  | Z75 | Problems related to medical facilities and other health care |
|  | J90 | Pleural effusion, not elsewhere classified |
|  | J69 | Pneumonitis due to solids and liquids |
|  | A49 | Bacterial infection of unspecified site |
| 11 | **Gastroenterology - Metabolic disorders and abdominal symptoms** | |
|  | K29 | Gastritis and duodenitis |
|  | D63 | Anemia in chronic diseases classified elsewhere |
|  | D64 | Other anemias |
|  | K92 | Other diseases of digestive system |
|  | Z85 | Personal history of malignant neoplasm |
|  | E87 | Other disorders of fluid, electrolyte and acid-base balance |
|  | D50 | Iron deficiency anemia |
|  | D46 | Myelodysplastic syndromes |
|  | E86 | Volume depletion |
|  | R11 | Nausea and vomiting |
|  | E83 | Disorders of mineral metabolism |
|  | K21 | Gastro-esophageal reflux disease |
|  | I10 | Essential (primary) hypertension |
|  | D61 | Other aplastic anemias and other bone marrow failure syndromes |
|  | R10 | Abdominal and pelvic pain |
|  | K56 | Paralytic ileus and intestinal obstruction without hernia |
|  | N17 | Acute kidney failure |
|  | D69 | Purpura and other hemorrhagic conditions |
|  | C79 | Secondary malignant neoplasm of other and unspecified sites |
| 12 | **Neurology - Stroke and emergencies involving the brain** | |
|  | G81 | Hemiplegia and hemiparesis |
|  | I63 | Cerebral infarction |
|  | R47 | Speech disturbances, not elsewhere classified |
|  | I64 | Stroke, not specified as haemorrhage or infarction |
|  | I69 | Sequelae of cerebrovascular disease |
|  | Z75 | Problems related to medical facilities and other health care |
|  | I61 | Nontraumatic intracerebral hemorrhage |
|  | G45 | Transient cerebral ischemic attacks and related syndromes |
|  | I67 | Other cerebrovascular diseases |
|  | I10 | Essential (primary) hypertension |
|  | I62 | Other and unspecified nontraumatic intracranial hemorrhage |
| 13 | **Pharmacology emergencies - Adverse drug effects of prescribed medications** | |
|  | T42 | Poisoning by, adverse effect of and underdosing of antiepileptic, sedative- hypnotic and antiparkinsonism drugs |
|  | X61 | Intentional self-poisoning by and exposure to antiepileptic, sedative-hypnotic, antiparkinsonism and psychotropic drugs, not elsewhere classified |
|  | T43 | Poisoning by, adverse effect of and underdosing of psychotropic drugs, not elsewhere classified |
|  | X41 | Accidental poisoning by and exposure to antiepileptic, sedative-hypnotic, antiparkinsonism and psychotropic drugs, not elsewhere classified |
|  | Y11 | Poisoning by and exposure to antiepileptic, sedative-hypnotic, antiparkinsonism and psychotropic drugs, not elsewhere classified, undetermined intent |
| 14 | **Toxicology - Emergencies and adversities due to substance abuse** | |
|  | F19 | Other psychoactive substance related disorders |
|  | F11 | Opioid related disorders |
|  | F14 | Cocaine related disorders |
|  | F10 | Alcohol related disorders |
|  | Z76 | Persons encountering health services in other circumstances |
|  | Y04 | Assault by bodily force |
|  | Z72 | Problems related to lifestyle |
|  | Z59 | Problems related to housing and economic circumstances |
|  | F13 | Sedative, hypnotic, or anxiolytic related disorders |
|  | Y09 | Assault by unspecified means |
| 15 | **Endocrinology - Diabetes and diabetic consequences** | |
|  | E14 | Unspecified diabetes mellitus |
|  | E11 | Type 2 diabetes mellitus |
|  | E10 | Type 1 diabetes mellitus |
|  | R73 | Elevated blood glucose level |
|  | H36 | Retinal disorders in diseases classified elsewhere |
|  | N08 | Glomerular disorders in diseases classified elsewhere |
|  | I79 | Disorders of arteries, arterioles and capillaries in diseases classified elsewhere |
|  | L97 | Non-pressure chronic ulcer of lower limb, not elsewhere classified |
|  | G63 | Polyneuropathy in diseases classified elsewhere |
|  | M86 | Osteomyelitis |
| 16 | **Gastroenterology and obstetrics - Conditions and symptoms of abdomen and pelvis** | |
|  | R10 | Abdominal and pelvic pain |
|  | N83 | Noninflammatory disorders of ovary, fallopian tube and broad ligament |
|  | Z33 | Pregnant state |
|  | N93 | Other abnormal uterine and vaginal bleeding |
|  | Z32 | Encounter for pregnancy test and childbirth and childcare instruction |
|  | N94 | Pain and other conditions associated with female genital organs and menstrual cycle |
|  | N73 | Other female pelvic inflammatory diseases |
|  | N39 | Other disorders of urinary system |
|  | K37 | Unspecified appendicitis |
|  | Z71 | Persons encountering health services for other counseling and medical advice, not elsewhere classified |
|  | G43 | Migraine |
|  | K35 | Acute appendicitis |
|  | N12 | Tubulo-interstitial nephritis, not specified as acute or chronic |
|  | R11 | Nausea and vomiting |
| 17 | **Cardiology - Chronic cardiovascular pathology** | |
|  | Y44 | Agents primarily affecting blood constituents |
|  | D68 | Other coagulation defects |
|  | R78 | Findings of drugs and other substances, not normally found in blood |
|  | Z92 | Personal history of medical treatment |
|  | I48 | Atrial fibrillation and flutter |
|  | I50 | Heart failure |
|  | I80 | Phlebitis and thrombophlebitis |
| 18 | **Trauma - Superficial injuries** | |
|  | V43 | Car occupant injured in collision with car, pick-up truck or van |
|  | S20 | Superficial injury of thorax |
|  | S30 | Superficial injury of abdomen, lower back, pelvis and external genitals |
|  | S19 | Other specified and unspecified injuries of neck |
|  | S10 | Superficial injury of neck |
|  | S39 | Other and unspecified injuries of abdomen, lower back, pelvis and external genitals |
|  | T00 | Superficial injuries involving multiple body regions |
|  | M54 | Dorsalgia |
|  | S40 | Superficial injury of shoulder and upper arm |
|  | V89 | Motor- or nonmotor-vehicle accident, type of vehicle unspecified |
|  | T14 | Injury of unspecified body region |
|  | S49 | Other and unspecified injuries of shoulder and upper arm |
|  | T09 | Other injuries of spine and trunk, level unspecified |
| 19 | **Infectious diseases and respirology - Acute and chronic disorders of airway and lungs** | |
|  | R06 | Abnormalities of breathing |
|  | J45 | Asthma |
|  | J18 | Pneumonia, unspecified organism |
|  | R05 | Cough |
|  | J44 | Other chronic obstructive pulmonary disease |
|  | J98 | Other respiratory disorders |
|  | J06 | Acute upper respiratory infections of multiple and unspecified sites |
|  | J40 | Bronchitis, not specified as acute or chronic |
|  | J20 | Acute bronchitis |
|  | R07 | Pain in throat and chest |
| 20 | **Toxicology - Alcohol-related emergencies** | |
|  | T51 | Toxic effect of alcohol |
|  | X45 | Accidental poisoning by and exposure to alcohol |
|  | Y15 | Poisoning by and exposure to alcohol, undetermined intent |
|  | X65 | Intentional self-poisoning by and exposure to alcohol |
|  | Y90 | Evidence of alcohol involvement determined by blood alcohol level |
| 21 | **Psychiatry - Schizophrenia and delusional disorders** | |
|  | F20 | Schizophrenia |
|  | F29 | Unspecified nonorganic psychosis |
|  | F25 | Schizoaffective disorders |
|  | F22 | Persistent delusional disorders |
|  | R44 | Other symptoms and signs involving general sensations and perceptions |
|  | R46 | Symptoms and signs involving appearance and behavior |
|  | F31 | Bipolar disorder |
|  | F99 | Mental disorder, not otherwise specified |
|  | Z59 | Problems related to housing and economic circumstances |
| 22 | **Pharmacology emergencies - Poisoning due to narcotics** | |
|  | T40 | Poisoning by, adverse effect of and underdosing of narcotics and psychodysleptics [hallucinogens] |
|  | X42 | Accidental poisoning by and exposure to narcotics and psychodysleptics [hallucinogens], not elsewhere classified |
|  | Y12 | Poisoning by and exposure to narcotics and psychodysleptics [hallucinogens], not elsewhere classified, undetermined intent |
|  | X62 | Intentional self-poisoning by and exposure to narcotics and psychodysleptics [hallucinogens], not elsewhere classified |
|  | F11 | Opioid related disorders |
| 23 | **Trauma - Injuries from contact with sharp instruments and machinery** | |
|  | S61 | Open wound of wrist, hand and fingers |
|  | W26 | Contact with knife, sword or dagger |
|  | W45 | Foreign body or object entering through skin |
|  | W25 | Contact with sharp glass |
|  | S91 | Open wound of ankle, foot and toes |
|  | W29 | Contact with other powered hand tools and household machinery |
|  | W49 | Exposure to other inanimate mechanical forces |
|  | W27 | Contact with nonpowered hand tool |
|  | S51 | Open wound of elbow and forearm |
|  | Z48 | Encounter for other postprocedural aftercare |
|  | W31 | Contact with other and unspecified machinery |
|  | W23 | Caught, crushed, jammed or pinched in or between objects |
| 24 | **Pharmacology emergencies - Poisoning due to hormones, cardiovascular drugs and other** | |
|  | X44 | Accidental poisoning by and exposure to other and unspecified drugs, medicaments and biological substances |
|  | T50 | Poisoning by, adverse effect of and underdosing of diuretics and other and unspecified drugs, medicaments and biological substances |
|  | X64 | Intentional self-poisoning by and exposure to other and unspecified drugs, medicaments and biological substances |
|  | Y14 | Poisoning by and exposure to other and unspecified drugs, medicaments and biological substances, undetermined intent |
|  | T45 | Poisoning by, adverse effect of and underdosing of primarily systemic and hematological agents, not elsewhere classified |
|  | T38 | Poisoning by, adverse effect of and underdosing of hormones and their synthetic substitutes and antagonists, not elsewhere classified |
|  | T46 | Poisoning by, adverse effect of and underdosing of agents primarily affecting the cardiovascular system |
|  | T49 | Poisoning by, adverse effect of and underdosing of topical agents primarily affecting skin and mucous membrane and by ophthalmological, otorhinolaryngological and dental drugs |
| 25 | **Pharmacology emergencies - Poisoning by pain killers and anti-inflammatory drugs** | |
|  | T39 | Poisoning by, adverse effect of and underdosing of nonopioid analgesics, antipyretics and antirheumatics |
|  | X60 | Intentional self-poisoning by and exposure to nonopioid analgesics, antipyretics and antirheumatics |
|  | X40 | Accidental poisoning by and exposure to nonopioid analgesics, antipyretics and antirheumatics |
|  | Y10 | Poisoning by and exposure to nonopioid analgesics, antipyretics and antirheumatics, undetermined intent |
| 26 | **Neurology - Epilepsy, seizures, brain lesions and other** | |
|  | G40 | Epilepsy and recurrent seizures |
|  | R56 | Convulsions, not elsewhere classified |
|  | G41 | Status epilepticus |
|  | Y46 | Antiepileptics and antiparkinsonism drugs |
|  | F44 | Dissociative and conversion disorders |
|  | D43 | Neoplasm of uncertain behavior of brain and central nervous system |
|  | G93 | Other disorders of brain |
|  | C71 | Malignant neoplasm of brain |
| 27 | **Trauma - Overexertion and injuries to the lower limb** | |
|  | X50 | Overexertion and strenuous or repetitive movements |
|  | S93 | Dislocation and sprain of joints and ligaments at ankle, foot and toe level |
|  | S99 | Other and unspecified injuries of ankle and foot |
|  | X59 | Exposure to unspecified factor |
|  | S83 | Dislocation and sprain of joints and ligaments of knee |
|  | S90 | Superficial injury of ankle, foot and toes |
|  | S82 | Fracture of lower leg, including ankle |
|  | T78 | Adverse effects, not elsewhere classified |
|  | X58 | Exposure to other specified factors |
| 28 | **Nephrology - Genitourinary disorders, disorders of prostate, other** | |
|  | T83 | Complications of genitourinary prosthetic devices, implants and grafts |
|  | R33 | Retention of urine |
|  | Z46 | Encounter for fitting and adjustment of other devices |
|  | R31 | Hematuria |
|  | Y84 | Other medical procedures as the cause of abnormal reaction of the patient, or of later complication, without mention of misadventure at the time of the procedure |
|  | N20 | Calculus of kidney and ureter |
|  | N39 | Other disorders of urinary system |
|  | N23 | Unspecified renal colic |
|  | N30 | Cystitis |
|  | B96 | Other bacterial agents as the cause of diseases classified elsewhere |
| 29 | **Neurology - Alzheimer’s diseases and dementia** | |
|  | G30 | Alzheimer's disease |
|  | F00 | Dementia in Alzheimer disease |
|  | F03 | Unspecified dementia |
| 30 | **Emergency medicine - Foreign body in eye, airway and other** | |
|  | W44 | Foreign body entering into or through eye or natural orifice |
|  | T15 | Foreign body on external eye |
|  | T18 | Foreign body in alimentary tract |
|  | T17 | Foreign body in respiratory tract |
|  | T16 | Foreign body in ear |
|  | H18 | Other disorders of cornea |
| 31 | **Emergency medicine - Complications of medical procedures** | |
|  | Y83 | Surgical operation and other surgical procedures as the cause of abnormal reaction of the patient, or of later complication, without mention of misadventure at the time of the procedure |
|  | T81 | Complications of procedures, not elsewhere classified |
|  | Y84 | Other medical procedures as the cause of abnormal reaction of the patient, or of later complication, without mention of misadventure at the time of the procedure |
|  | T82 | Complications of cardiac and vascular prosthetic devices, implants and grafts |
| 32 | **Environmental exposures - Exposure to heat and light** | |
|  | X30 | Exposure to excessive natural heat |
|  | T67 | Effects of heat and light |
| 33 | **Environmental exposures - Exposure to cold/hypothermia** | |
|  | X31 | Exposure to excessive natural cold |
|  | T35 | Frostbite involving multiple body regions and unspecified frostbite |
|  | T68 | Hypothermia |
| 34 | **Environmental exposures - Bee, wasp and hornet stings** | |
|  | T63 | Toxic effect of contact with venomous animals and plants |
|  | X23 | Contact with hornets, wasps and bees |
| 35 | **Infectious diseases - Viral conjunctivitis** | |
|  | B30 | Viral conjunctivitis |
|  | H13 | Disorders of conjunctiva in diseases classified elsewhere |
| 36 | **Trauma - Assault and intentional injury** | |
|  | X99 | Assault by sharp object |
|  | S21 | Open wound of thorax |
|  | S11 | Open wound of neck |
|  | S41 | Open wound of shoulder and upper arm |
|  | T01 | Open wounds involving multiple body regions |
|  | S27 | Injury of other and unspecified intrathoracic organs |
|  | X78 | Intentional self-harm by sharp object |
|  | S31 | Open wound of abdomen, lower back, pelvis and external genitals |
|  | S51 | Open wound of elbow and forearm |
| 37 | **Pharmacology emergencies - Adverse reactions to antibiotics and other drugs** | |
|  | Y40 | Systemic antibiotics |
|  | L27 | Dermatitis due to substances taken internally |
|  | T88 | Other complications of surgical and medical care, not elsewhere classified |
|  | T78 | Adverse effects, not elsewhere classified |
|  | Y57 | Other and unspecified drugs and medicaments |
|  | R21 | Rash and other nonspecific skin eruption |
|  | L50 | Urticaria |
|  | X58 | Exposure to other specified factors |
| 38 | **Trauma - Adult and child abuse and sexual assault** | |
|  | T74 | Adult and child abuse, neglect and other maltreatment, confirmed |
|  | Y05 | Sexual assault by bodily force |
|  | Y07 | Perpetrator of assault, maltreatment and neglect |
| 39 | **Environmental exposures - Burn and chemical poisoning** | |
|  | T26 | Burn and corrosion confined to eye and adnexa |
|  | W89 | Exposure to man-made visible and ultraviolet light |
|  | X49 | Accidental poisoning by and exposure to other and unspecified chemicals and noxious substances |
|  | X08 | Exposure to other specified smoke, fire and flames |
|  | T54 | Toxic effect of corrosive substances |
|  | T65 | Toxic effect of other and unspecified substances |
| 40 | **Gynecology - Infections of reproductory organs and other** | |
|  | N77 | Vulvovaginal ulceration and inflammation in diseases classified elsewhere |
|  | B37 | Candidiasis |
|  | A60 | Anogenital herpesviral [herpes simplex] infections |
|  | N76 | Other inflammation of vagina and vulva |
| 41 | **Pharmacology emergencies - Poisoning due to drugs acting on autonomic nervous system** | |
|  | X63 | Intentional self-poisoning by and exposure to other drugs acting on the autonomic nervous system |
|  | T44 | Poisoning by, adverse effect of and underdosing of drugs primarily affecting the autonomic nervous system |
| 42 | **Environmental exposures - Toxic effect of gases, fumes and vapors** | |
|  | T59 | Toxic effect of other gases, fumes and vapors |
|  | X47 | Accidental poisoning by and exposure to other gases and vapours |
|  | X09 | Exposure to unspecified smoke, fire and flames |
| 43 | **Environmental exposures - Exposure to electrical current** | |
|  | W87 | Exposure to unspecified electric current |
|  | T75 | Other and unspecified effects of other external causes |
